# Whole-genome sequencing of recurrent neuroblastoma reveals somatic mutations that affect key players in cancer progression and telomere maintenance

**DOI:** 10.1038/s41598-020-78370-7

**Published:** 2020-12-31

**Authors:** Susanne Fransson, Angela Martinez-Monleon, Mathias Johansson, Rose-Marie Sjöberg, Caroline Björklund, Gustaf Ljungman, Torben Ek, Per Kogner, Tommy Martinsson

**Affiliations:** 1 Department of Laboratory Medicine, Institute of Biomedicine, University of Gothenburg, Box 445, 405 30 Gothenburg, Sweden; 2 Science for Life Laboratory, Clinical Genomics, Gothenburg, Sweden; 3 Department of Pediatrics, Umeå University Hospital, Umeå, Sweden; 4 Department of Women’s and Children’s Health, Children’s University Hospital, University of Uppsala, Uppsala, Sweden; 5 Children’s Cancer Center, Queen Silvia Children’s Hospital, Sahlgrenska University Hospital, Gothenburg, Sweden; 6 Department of Women’s and Children’s Health, Karolinska Institutet, Stockholm, Sweden

**Keywords:** Cancer, Genetics, Oncology

## Abstract

Neuroblastoma is the most common and deadly childhood tumor. Relapsed or refractory neuroblastoma has a very poor prognosis despite recent treatment advances. To investigate genomic alterations associated with relapse and therapy resistance, whole-genome sequencing was performed on diagnostic and relapsed lesions together with constitutional DNA from seven children. Sequencing of relapsed tumors indicates somatic alterations in diverse genes, including those involved in RAS-MAPK signaling, promoting cell cycle progression or function in telomere maintenance and immortalization. Among recurrent alterations, *CCND1*-gain, *TERT*-rearrangements, and point mutations in *POLR2A*, *CDK5RAP*, and *MUC16* were shown in ≥ 2 individuals. Our cohort contained examples of converging genomic alterations in primary-relapse tumor pairs, indicating dependencies related to specific genetic lesions. We also detected rare genetic germline variants in DNA repair genes (e.g., *BARD1*, *BRCA2*, *CHEK2*, and *WRN*) that might cooperate with somatically acquired variants in these patients with highly aggressive recurrent neuroblastoma. Our data indicate the importance of monitoring recurrent neuroblastoma through sequential genomic characterization and that new therapeutic approaches combining the targeting of MAPK signaling, cell cycle progression, and telomere activity are required for this challenging patient group.

## Introduction

Neuroblastoma (NB) is an aggressive pediatric malignancy originating from the sympathetic nervous system. It is characterized by a diverse clinico-biological phenotype, with nearly half of all patients having a high-risk profile. Despite recent improvements in multimodal treatment, including high-dose induction chemotherapy, followed by surgery and myeloablative therapy with autologous stem cell rescue and local radiotherapy, maintenance treatment with retinoic acid, as well as immunotherapy using anti-GD2, the long-term survival of children diagnosed with high-risk NB remains below 50% at 5 years from treatment^1,2^. Although initial response to treatment is seen in most patients, a significant portion will subsequently progress with lesions resistant to standard therapy, and for NB patients with relapsed or refractory metastatic disease, survival is below 10%^2^. Genetic risk factors previously identified in primary NB include larger chromosomal aberrations, such as amplification of the *MYCN* oncogene, 11q-deletion, 1p-deletion, and 17q-gain^3^, as well as activating mutations in *ALK* encoding anaplastic lymphoma kinase^4–8^. Additional genomic alteration that recently have been associated to inferior outcome are distal 6q-deletion^9,10^, 19p-deletion and 1q-gain^11,12^ and aberrations connected to *ATRX*, *TERT*, *TP53* or RAS^13^. Analysis of primary NB tumors indicates a relative paucity of recurrent somatic alterations, with mutations in *ALK* being most frequent^14–17^. However, treatment-resistant tumors will likely have novel molecular and genetic aberrations different from those present at time of diagnosis, either induced by or selected for during treatment. Previous studies of relapsed NB indicate that alterations associated with activation of the ALK-RAS-MAPK pathway^18,19^ or mesenchymal transition^20^ are enriched in relapsed samples. To further investigate the genetic mechanisms linked to recurrent disease, alterations between seven triplicate samples of primary tumor, relapsed or refractory tumor, and constitutional DNA were investigated using whole-genome sequencing (WGS).

## Results

### Clinical background and patient characteristics

We performed WGS on the genomes of triplicate samples of primary and relapsed/refractory NB tumors and lymphocytes from seven patients. All tumors were high-stage, high-risk tumors with metastatic disease at time of diagnosis. The median age at time of diagnosis was 50 months (range 17–118 months), while the median time from diagnosis to relapse or progression was 26 months (range 12–71 months). All subjects were diagnosed between 2012 and 2017 and initially treated according to the HR-NBL1/SIOPEN protocol and thus, underwent similar treatment consisting of induction chemotherapy (rapid COJEC ± TVD), local therapy (surgery and local irradiation), high-dose chemotherapy (BuMel) with autologous stem cell rescue, and maintenance therapy (retinoids). Six of the seven patients had recurrence after complete clinical remission after completed therapy and one patient experienced disease progression during treatment (Table 1). None of these patients had received any additional treatment using targeted therapy before the time of relapse/progression.

**Table 1:** Clinical characteristics of patients.

	NB60R6	NB67R5	NB67R9	NB63R4	NB59R9	NB53R2	NB69R8
Gender	F	M	M	M	M	F	F
Risk stratification	HR	HR	HR	HR	HR	HR	HR
INRGSS/INSS	M/4	M/4	M/4	M/4	M/4	M/4	M/4
Genomic profile	11q-del	17q-gain	11q-del	MNA/11q-del	12q-amp	11q-del	Other segmental
Age at diagnosis, months	50	83	36	36	63	17	118
Time to relapse, months	31	26	12	24	27	71	24
Outcome	DOD	DOD	DOD	NED	AWD	DOD	NED
**At diagnosis**							
Clinical background	Metastatic spreading with hyperplasia in liver	Large thoracic-abdominal tumor with extensive metastatic spread in bone and bone marrow	Paravertebral tumor in thorax with metastatic spread to orbital regions and para-aortal lymph nodes	Primary tumor in right adrenal gland with extensive metastatic spread in retroperitoneal and pelvic bone, left femur, and bone marrow	Large abdominal mass with retroperitoneal spread and multiple metastases in liver and lung	Tumor in left kidney with axillary spread to abdomen and metastatic disease in lung, scull bone, and bone marrow	Abdominal tumors in left adrenal gland and a large metastasis in proximity to pancreas; no metastatic spread to bone or bone marrow
Material subject to analysis	Metastatic lesion	Primary tumor of paravertebral mass on left side of thorax	NA	Primary tumor	Primary tumor	Primary tumor	Primary tumor
First line of treatment	HR-NBL1, COJEC + TVD + Surgery + BuMel + Local irr + Retinoids	HR-NBL1, COJEC + 2 TVD	HR-NBL1, COJEC, Surgery, high-dose BuMel, Local irr	HR-NBL1, COJEC + Surgery + BuMel + Local irr + Retinoids	HR-NBL1, COJEC + Surgery + BuMel + Local irr + Retinoids	HR-NBL1, COJEC + Surgery + BuMel + Local + scull irr + Retinoids	HR-NBL1, COJEC + Surgery + Local irr + Retinoids
Response to first line of treatment	Delayed response to COJEC, hence given TVD; complete clinical remission after completed therapy	Mixed response, partial response in primary tumor; progression with new metastatic lesions during treatment; no clearance of bone marrow	Very good partial remission; complete response in bone marrow; radical surgery of primary tumor	Good response to rapid COJEC; complete clinical remission after completed therapy	Good response to rapid COJEC; complete clinical remission after completed therapy	Good response to rapid COJEC; complete clinical remission after completed therapy	Partial response to rapid COJEC; complete clinical remission after completed therapy
**At relapse**							
Clinical background	After complete clinical remission, during follow-up with delayed examinations for administrative reasons, symptoms of relapsing metastatic disease occurred: pain, enlarged liver, decreasing blood counts	After progression during treatment, change of strategy to palliative treatment based on RIST-protocol, stable disease for 19 months; at evaluation, increased activity at metastatic lesions	Metastatic relapse during retinoid therapy; local surgery for biopsy; medical treatment with celecoxib + RIST protocol; limited response and then clinical progression	Detected at routine follow-up with MRI and MIBG	Detected at routine follow-up imaging with CT and MRI	Pain in her right foot led to MRI that showed multiple suspicious metastases in skeleton and bone marrow; confirmed by MIBG	Metastatic disease in mediastinal lymph nodes, lungs and skeleton
Material subject to analysis	Metastatic lesion	Metastatic lesion, thorax left side	Metastasis to skull bone	Metastatic lesions, lymph gland and bone marrow	Metastatic lesion, lung	Metastatic lesion, bone	Metastatic lesion, mediastinum
Second line of treatment	RIST + celecoxib	Trametinib (1 month)	RIST + celecoxib	Valproic acid, RIST	Ribociclib, TEMIRI. RT. RIST + celecoxib; etoposide	RIST + celecoxib	TEMIRI 2 courses; lorlatinib
Response to second line of treatment	Poor. Progressive disease	Poor. Progressive disease	Poor. First stable disease, then progression	Very good: complete clinical remission after 2, 3 years of treatment; continues with valproic acid	Poor. Progressive disease	Good: complete clinical remission after 6 months of treatment. New relapse after 15 months	No response to TEMIRI; complete response to lorlatinib

F, female; M, male; INRGSS, International Neuroblastoma Risk Group Staging System; INSS, International Neuroblastoma Staging System; DOD, dead of disease; NED, no evidence of disease; AWD, alive with disease; MNA, MYCN-amplification; irr, irradiation; CR, complete remission according to INRC; COJEC, cisplatin [C], vincristine [O], carboplatin [J], etoposide [E], and cyclophosphamide [C]; BuMel, busulfan [Bu] and melphalan hydrochloride [Mel]; TVD, topotecan [T], vincristine [V], and doxorubicin [D]; RIST, rapamycin [R], irinotecan [I], dasatinib [S] and temozolomide [T]; TEMIRI, temozolomide [TEM] and irinotecan [IRI]; HR-NBL1 (NCT01704716, clinicaltrials.gov); NA, not available.

### Constitutional alterations in cancer-predisposing genes

To detect possible genetic predisposition to NB, constitutional DNA was analyzed, focusing only on variants in 565 genes with established associations with sporadic and/or hereditary cancer (Supplemental Table 2). This analysis did not detect any activating point mutations in hot-spot regions of *ALK* or loss-of-function variants in *PHOX2B*, the two genes mainly connected to NB predisposition. However, a novel *ALK* frameshift variant (i.e., ALK p.V1471fs*6) of unknown significance was detected in one patient. This variant is predicted to result in a truncated protein that retains the kinase domain but lacks a portion of the C-terminal domain containing multiple phosphorylation sites crucial for ALK functions including tyrosine residue 1604. Thus, it is unlikely that this variant has the transforming potential seen for of the *ALK* hot-spot mutations common in NB. Three of the seven patients had germline variants likely to affect the function of DNA repair genes. These include a nonsense mutation in *BARD1* (p.Q545), a missense variant in *WRN* (p.S1141L) affecting an ATM phosphorylation site important for WRN protein function, and two rare missense variants in *CHEK2* (p.I157T) and *BRCA2* (p.R2991C), respectively, detected in one patient. These variants and other variants associated with loss-of-function or predicted to be damaging by SIFT and PolyPhen2 are summarized in Table 2.

**Table 2:** Possibly damaging/pathogenic germline variants.

ID	Chr	Pos	Ref	Var	Gene	Protein	SIFT	PP2	gnomAD frequency	COSMIC	dbSNP
NB60R6	3	41266655	G	A	CTNNB1	p.R151H	Damaging	Possibly damaging	0.003	2987951	200968230
NB60R6	14	81554373	G	A	TSHR	Splice Site Loss					751583621
NB67R5	3	71021260	C	T	FOXP1	p.W534*			0.005		140157154
NB67R9	13	32953904	C	T	BRCA2	p.R2991C		Benign	0.001	1366489; 1366490	751787816
NB67R9	18	61006050	TTTG	–	KDSR	p.Q253fs*24			0.002		764767580
NB67R9	22	29121087	A	G	CHEK2	p.I200T; p.I157T	Tolerated	Possibly damaging	0.492	3693990	17879961
NB67R9	22	40814864	C	T	MRTFA (MKL1)	p.M526I; p.M476I; p.M561I	Damaging	Possibly damaging		4104397	
NB67R9	22	41543910	A	G	EP300	p.Q734R	Tolerated	Possibly damaging	0.008		779657418
NB63R4	2	29416543	G	–	ALK	p.V1471fs*6					
NB63R4	2	215610566	G	A	BARD1	p.Q545*; p.Q94*; p.Q564*; p.Q113*			0.002		587780021
NB59R9	8	31004607	C	T	WRN	p.S1141L	Tolerated	Benign	0.08		139323683
NB59R9	14	45665613	G	A	FANCM	p.R1834H; p.R1860H	Damaging	Probably damaging	0.006	955827	776506025
NB59R9	17	37882080	T	C	ERBB2	p.I949T; p.I934T; pI949T	Damaging	Probably damaging			
NB53R2	5	158135077	C	T	EBF1	p.V545M; p.V493M; p.V521M; p.V544M; p.V552M; p.V420M; p.V553M	Damaging	Possibly damaging	0.004	6651137	752613065
NB69R8	12	133245307	T	A	POLE	p.D620V; p.D647V	Damaging	Possibly damaging			1060500884
NB69R8	10	70333991	TGT	–	TET1	p.V635del					903550402

### Overall comparison of somatic variants between time of diagnosis and time of recurrence

**Figure 1:**
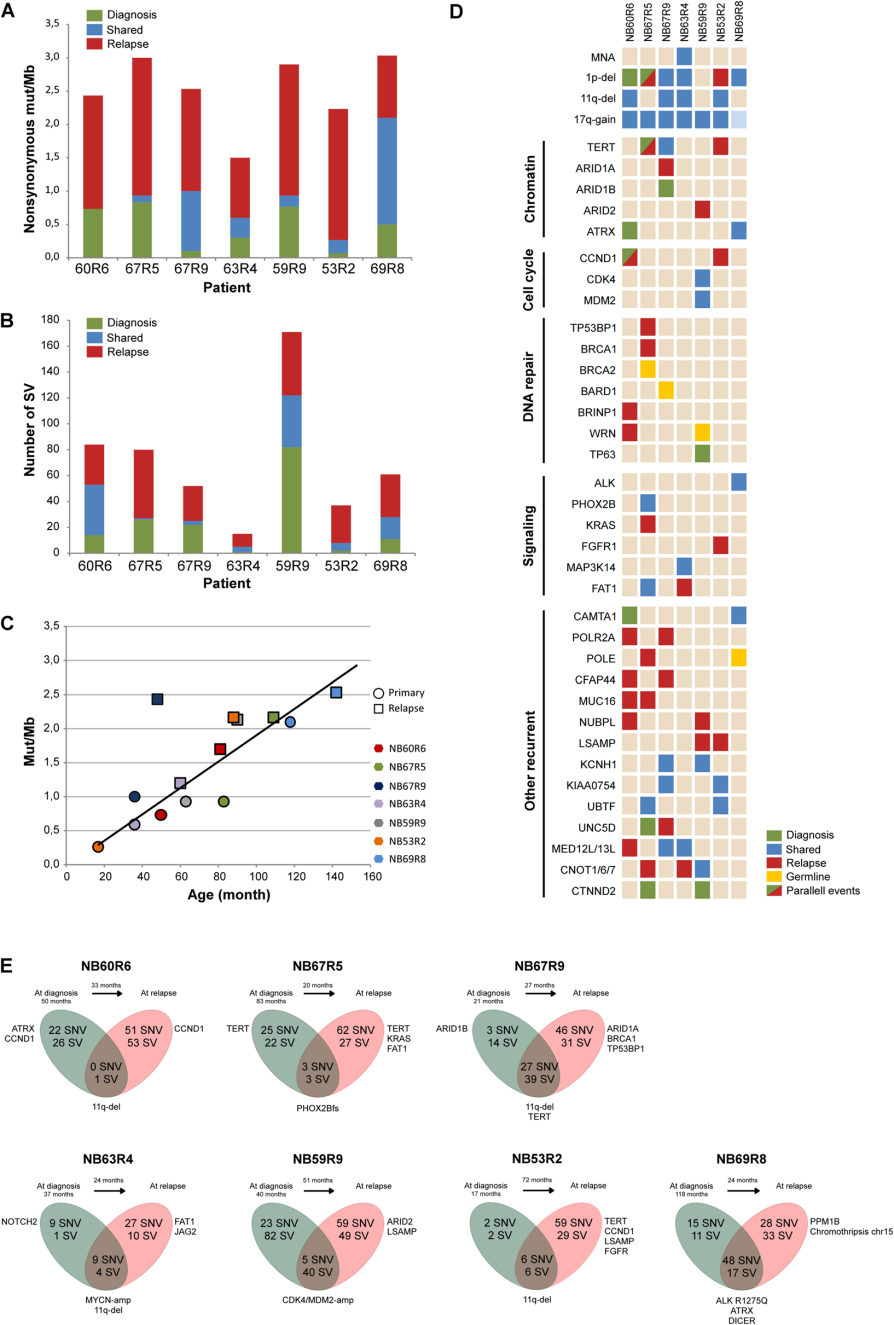
Mutational distribution of primary and relapsed NBs. (**A**) The number of non-silent somatic mutations per megabase identified in seven primary tumors and their corresponding relapse tumors. Mutations shared by primary and relapse tumors are shown in blue, mutations unique to primary tumors are shown in green, and mutations unique to relapse tumors are shown in red. (**B**) The total number of somatic structural variants identified in the seven primary tumors and their corresponding relapse tumors; color scheme as in (**A**). (**C**) Number of nonsynonymous somatic mutations per megabase in primary and relapse tumors in relation to age at time of diagnosis and age at time of relapse/progression. (**D**) Recurrent and other biological relevant copy number alterations and SNVs detected through WGS. (**E**) Case-by-case overview of somatic SNVs and SVs in primary and relapsed tumors with biologically relevant mutated genes and/or genomic alterations indicated.

In total, 529 nonsynonymous somatic SNVs were detected in the analyzed tumors (Table 3 and Supplemental Table 3), with an average of 28 SNVs (range 8–63) in primary tumors, increasing to an average of 61 SNVs in relapsed tumors (range 51–76), corresponding to 0.7 and 2.1 variants per megabase of the coding genome, respectively. The mutational burden at time of relapse/progression was significantly higher than at time of diagnosis (*p* = 0.002) (Fig. 1A,B). Overall, the mutational load was in the range of that previously reported in NB. The median number of somatic SNVs shared by each primary and relapse/refractory pair was 14 (range 0–40), corresponding to 50% of all mutations detected in primary tumors and to 23% of all mutations detected in relapsed/refractory tumors. However, for patient NB60R6, for whom the investigated biopsy specimens were from a metastasis at time of diagnosis and a metastasis at time of relapse, no somatic nonsynonymous SNV shared by the two samples could be detected, not even after including mutations below the 10% variant allele fraction.

**Table 3:** Overview of nonsynonymous somatic SNVs, either shared or unique to primary of relapsed tumors, respectively; truncating SNVs indicated in bold and tumorigenic SNVs indicated in italic.

At diagnosis		Common		At relapse		
**NB60R6**						
ABCA9 (L117F)	ITGA2B (E673*)			ABCB1 (R148G)	EIF4A3 (A280V)	PYHIN1 (P188R; P197R)
AIM1 (K1595I)	KCNU1 (N1010S)			ABCC2 (Q1179E)	FADS6 (M279I)	REG1A (W149*)
BFSP1 (E276K)	NCKAP5 (C1020F)			ANKRD31 (Q1669H)	FAM107B (S93*)	SETBP1 (K419E)
C3 (E1258Q)	NEURL4 (F1392fs*7)			ANO3 (V428A)	***FLT1 (L1150I)***	SLC27A5 (R110P)
CARNS1 (P212L;)	SAMD5 (E121D)			ASB10 (R138Q)	FN3KRP (S58R)	SLC35B4 (G55A)
CCDC157 (Q22H)	**SLC2A12 (L66del)**			C1orf159 (P240L)	GJA3 (H95N)	SNTG1 (G411*)
CGNL1 (R343W)	SOGA1 (G1567V)			C1orf159 (R239fs*42)	GTF3C1 (V1421M)	SS18 (P336T)
DYSF (R1698C)	SORCS1 (T435fs*32)			CDK5RAP1 (N233D)	INTS7 (Q530H)	TRIP6 (H392R)
FZD5 (Q472K)	SP7 (P102T)			CFAP44 (I156V)	LINGO1 (V237L)	TRPM2 (F1020V)
GRAMD1A (C170F)	TTI1 (Q693K)			CITED1 (H112N)	LRRIQ1 (T1359K)	UGGT1 (Q967*)
IMPG2 (A691E)	ZNF676 (W432L)			CLSTN2 (A732G)	MEGF10 (A826fs*4)	USP11 (V425L)
				COL11A2 (P1460LL)	MUC16 (V2550M)	USP9X (L510R)
				CREG2 (R88Q)	MUC5B (A665V)	***WRN (T642I)***
				DDX60 (L29S)	OR9G4 (H259N)	ZNF25 (R34S)
				DLGAP1 (D470E)	P4HA1 (D222N)	ZNF391 (G22R)
				DNAH17 (R2788G)	POLR2A (R292C)	ZNF618 (K535M)
				DOCK2 (D1562fs*5)	PRKG2 (K67N)	ZNF814 (V204G)
**NB67R5**						
ABCB5 (A375S)	MUC19 (G5940E)	GMPS (A547G)		A1CF (G136E)	FSCN3 (D412Y)	OSR2 (L111S)
ATP8B3 (Y116F)	PCLO (D2180H)	***PHOX2B (G230fs)***		ABCA7 (D250N)	GDA (D356Y)	PALM (E29*)
C8orf4 (R79P)	PNPLA3 (Q370*)	***FAT1 (E1027D)***		ACOXL (R95S)	GFRA1 (Q111fs*5)	**PDGFRB (E15K)**
CADPS2 (T1245S)	RELN (M934V)			ACTRT2 (F2I)	GIMAP2 (S83P)	PGLYRP4 (P207T)
CREB1 (L179V)	SPTBN2 (K2304N)			ALDH1L2 (D483H)	GIMAP6 (G205R)	POLE (K182N)
CSF2RB (V449M)	TAS2R9 (F280L)			ANKRD20A4 (G772E)	GTF3C5 (L132F)	SIGLEC10 (K329N)
CXorf21 (T82K)	TUBB8 (Y50H)			AP2A2 (V865F)	HYDIN (G5011R)	SMIM35 (V44L)
EVPL (V155M)	UNC5D (W309R)			AP3B1 (L47P)	ISOC1 (G46D)	STX12 (R97C)
EXPH5 (E1145*)	WAPL (C713F)			ATAD5 (T253P)	KIAA2012 (N622K)	TDRD5 (A386V)
GLI2 (T607K)	YAP1 (R233Q)			**B4GALT2 (Splice site)**	***KRAS (K117N)***	TDRD6 (Q285K)
GPR162 (R411P)	ZFAT (E26V)			C19orf53 (G28D)	**LIPG (Splice site)**	TG (L2758F)
LRRC8E (D791G)	ZNF516 (Q46K)			C6orf222 (P476T)	MUC16 (G10463R)	TG (S2757R)
MUC19 (G3093V)				CAPS (M1I)	MUC6 (A240D)	TMEM131L (A1272V)
				CEBPA (P14R)	MYH15 (I89T)	TRIO (S2476C)
				CGN (E402V)	MYH6 (A1685D)	TUBB8 (C266R)
				**CHAT (R659fs?)**	MYH6 (A1685P)	VGF (V476A)
				CHODL (P174S)	MYO16 (P1661L)	VPS41 (G172W)
				CSRNP1 (E248D)	NECTIN2 (F323L)	ZNF316 (A788V)
				EIF4ENIF1 (S777Y)	NOMO3 (E603V)	**ZNF638 (Splice site)**
				ENDOV (S93W)	NOP14 (N270Y)	ZNF800 (D208G)
				ENTPD1 (D3E)	NR5A2 (M175L;)	ZNF878 (K258Q)
**NB67R9**						
AUTS2 (p.L1031fs*21)		ADRA2B (P325H)	PHLDB3 (Q178K)	ADGRV1 (P934T)	GFAP (A322D)	PSG2 (H12N)
FAM83B (p.G304V)		ANK2 (A2042D)	PSD3 (R63I)	***ARID1A (G388R)***	GORASP1 (E414V)	RAD51AP2 (I681N)
**ODF2 (Splice site)**		ARHGAP23 (D688N)	PXDN (A323S)	BAZ1B (E889D)	HS6ST1 (G162C)	RHAG (A184D)
		CFH (Q1076E)	RFPL4A (V155L)	***BRCA1 (N1045D)***	IGF2R (S1158I)	**SCN9A (Splice site)**
		DNAH3 (N634K; N694K)	RNF145 (M153V)	CCDC160 (E284V)	INTS5 (E124K)	TMEM102 (D100G)
		ENPP3 (N741C)	SCAP (R492fs*81)	CCDC57 (L263F)	IQGAP3 (A423E)	TMPRSS11E (L96*)
		GBP4 (G445*)	SETSIP (D72A)	CCT6B (N37H)	MORN1 (E119Q)	TP53BP1 (A1853V)
		GLIS3 (P80R; P235R)	SP2 (F560L)	CDH18 (K66E; K289E)	MRPS34 (D110V)	TSC22D2 (E435D)
		GRIK4 (A913G)	TTLL6 (E176K)	CFAP44 (P480T)	MYRIP (G261E)	TXNDC5 (A224G)
		GUCY2C (S21P)	VPS45 (Q252K)	CYBRD1 (F139I)	NBR1 (P348fs*2)	UNC5D (A245P)
		HTR2A (A390D; A306D)	ZFYVE28 (C529F)	DGKI (Splice site)	**NTSR2 (L221Q)**	VCAM1 (D329Y)
		KIAA0100 (V497F)		DNAH11 (T308K)	ORC1 (S832R; S837R)	VCAN (A1406T)
		KIAA0754 (F1145S)		DZANK1 (D739H)	PFKL (S255fs*3)	ZNF229 (D38Y)
		MAP2K7 (P286R)		E2F8 (L266S)	POLR2A (G156V)	ZNF519 (N156S)
		NLRP4 (E54*)		FTCD (K318M)	PROCA1 (S271I)	
		PCDH15 (S1841*)		FTSJ3 (R846Q)	PRR12 (G1678W)	
**NB63R4**						
CLCNKA (A287V)		C2orf27A (P186S)		AHNAK2 (P444S)	GLT6D1 (G257R)	RGS8 (*181Y; )
NOTCH2 (G1339R)		CCDC74B (P135S; P201S)		ARHGEF38 (Q62K)	GRIN3A (G618D)	RP1 (T1618A)
FAM180A (L65I)		CDHR2 (L842M)		CASKIN2 (C60Y)	INTS6L (Y557C)	S1PR5 (P10T)
SLC38A4 (V502F)		CXorf40A/CXorf40B (C53W)		CD1C (S35*)	JAG2 (G821C)	SAMD11 (G650W)
ANKRD40 (R177W)		DPYS (P222R)		CNOT7 (L58I)	**KIAA1549 (Splice site)**	SORL1 (R724I)
TCHP (Q222K)		***MAP3K14 (V568M)***		ERCC2 (K101M; K77M)	MUC17 (P375H)	SOX7 (R102C)
DNAAF2 (R609I)		MED12L (Q1307K)		***FAT1 (N40S)***	MVK (A282T)	SPATA5L1 (P386fs*11)
FOXK2 (A25S)		OR56A1 (A164S)		FER1L6 (H1352N)	PRR12 (K964N)	UPF1 (G61D)
FOXK2 (G26delinsPP)		SAMD9 (L70I)		FOXN4 (Q357fs*49)	PSG5 (A22E)	USP6 (H191N)
**NB59R9**						
ACIN1 (E142*)	TNF (A221D)	FGF4 (G185A)		A2M (F1431L)	IFRD2 (C313F)	RNF145 (M141T)
AKAP8 (E33K)	ZNF845 (D471E)	FSCN1 (E215Q)		ACSS2 (G159V)	IL12RB2 (S717Y)	RPS7 (K155N)
ALAS2 (S474R)	ZNF879 (G462V)	H3F3A (A115G)		***ARID2 (Q1124H)***	ILF3 (A599P)	SCN4A (W762*)
CCT6B (L318I)		OPHN1 (D426*)		C3 (S1302I)	ISCA1 (E103D)	SCN8A (G472fs*53)
CLDN6 (Y165C)		TTC17 (D154Y)		CARNS1 (A406E)	KRT31 (C411F)	SCN8A (G472V)
***EYA2 (A93S)***				CCDC141 (L698*)	LRRC39 (Splice site)	SIGLEC1 (G1658A)
GPR142 (R168C)				CCDC170 (K158R)	MRC2 (S1084fs*46)	SLC4A1 (E272V)
GRIA3 (N343S)				CHRNB1 (F290V)	MS4A12 (D146G)	SND1 (K71N)
INSR (A1233S)				CKMT2 (A192V)	MYBPC2 (W979C)	SPTA1 (A2341D)
KTN1 (A1235D)				CTDP1 (E826D)	NEUROG1 (A190D)	SUSD1 (A383S)
MGAM (E513*)				CYLD (A439D)	NLRP9 (E358K)	TBC1D19 (Y322C)
MUC7 (Q332H)				DDX53 (M361I)	**NUBPL (Splice site)**	TBC1D30 (Q494H)
MYH2 (D515N)				DOHH (A46S)	OSMR (G464R)	THRAP3 (P276A)
NDUFAF7 ()				DRP2 (K906N)	PCDHGA8 (V470F)	TMEM132B (Q311H)
PHF6 (Y313C)				FAHD1 (G116R)	PCDHGB1 (A697fs*2)	TRAF3 (K55N)
PIGQ (W639*)				GAL3ST2 (A262V)	PF4 (E59K)	WBSCR22 (D104Y)
PKD1 (F3056L)				GIPC1 (Q116H)	PLA2G3 (R67M)	WDFY3 (G2681*)
RTN1 (A744E)				GLRA4 (L259V)	PSME4 (F1728S)	XIRP2 (K2454T; K2676T)
SGK3 (A92E)				GNG7 (A32S)	PWWP2A (E255D)	ZNF416 (G528R)
SIN3A (L508V)				GPR68 (V96L)	RAB3GAP2 (K579N)	
**NB53R2**						
CHAC1 (R211Q)		CCDC66 (S563P)		ACSM2B (E185*)	FAM104B (Y90F)	MSI1 (A79S)
SMU1 (A26T)		GJA8 (L29I)		ADCY5 (A532V)	FAM172A (R181L)	NEBL (A697V)
		KIAA0754 (T1014A)		ANKRD6 (E617K)	FBN3 (G56R)	OR2L13 (F11L)
		LMTK3 (R1330W)		AUTS2 (G178V)	FBXO22 (E115*)	OR4A47 (S290Y)
		PSD4 (G220V)		BBX (E302Q)	***FGFR1 (W646S)***	OR4K17 (P198H)
		WASHC2A/WASHC2C (L569S)		BMP8B (A282T)	GOT1 (Q287K)	OR5T1 (L4F)
				CACNA1D (S228R)	GPANK1 (G271*)	OTX1 (A91V)
				CCDC138 (L260*)	GPR119 (R287*)	PCNA (S186G)
				CEP290 (K1227I)	HEATR6 (Q958H)	PZP (N970K)
				CLDN34 (N141K)	HLA-DOB (L193H)	REPS2 (S548C)
				CLEC4E (F138fs*21)	HYAL1 (F204L; F22L)	RNF123 (V555F)
				COL4A3 (G1140C)	IGSF22 (L1160fs*38)	SLC35F6 (L15V)
				CRAMP1 (A1120E)	INPP5F (G290E)	SLC5A10 (A229D)
				CXorf67 (E25K)	KCNH2 (A244P)	SNRPB2 (K111T)
				DGKK (W30*)	KIAA0368 (D404G)	SP110 (K51N)
				DSG3 (V423F)	KIDINS220 (M940V)	SULT1E1 (H107N)
				EHMT2 (R416Q)	KIF18B (W742L)	TXNDC11 (V335F)
				ELOVL7 (G94D)	KLHL7 (H363N)	VWC2 (L228F)
				ENOX2 (A222S)	LNX1 (F28L)	ZC3H12B (G541R)
				ESPL1 (Q1674*)	LYPD3 (A261V)	
**NB69R8**						
ABLIM3 (H114Y)		ABCA7 (N1518K)	NIPAL1 (L146M)	AACS (S513F)	TIGD1 (S32*)	
ABLIM3 (L107V)		ACOX3 (S135F)	NOXA1 (A195E)	ANGPT4 (N179K)	TPM3 (E141A)	
AKAP12 (R210C)		ADAM22 (S427I)	OR56A1 (P292T)	ARHGAP29 (P463R)	TRABD2B (R47H)	
ATP10B ( L1208V)		AKR1D1 (E136K)	**PLEKHG1 (Splice site)**	ARHGEF26 (T635P)	ZNF292 (Q897L)	
FBN3 (P2474S)		***ALK (R1275Q)***	PNKP (V443I)	B4GALNT3 (P916S)		
GALNT17 (K268R)		APEX2 (L317F)	PRAMEF2 (F430L)	CA6 (W35R)		
MS4A3 (D38Y)		BMS1 (M180I)	PRR23C (E74Q)	CDCA8 (R144S)		
MYO10 (L538V)		CCDC88B (P582H)	RELN (T468P)	CYP4A22 ( D432E)		
NOTCH3 (L1032F)		CEP120 (L919F)	RXFP1 (A426E)	KRT35 (R317H)		
OR5AS1 (N128D)		CRX (L299M)	SH3RF1 (R446H)	MBD5 (G10E)		
SACS (M3765V)		CUX1 (Q835*)	SI (Q153K)	NCAN (E877V)		
SLC5A7 (G81S)		CXCL12 (K104E)	SLC1A1 (T364K)	**NUP205 (Splice site)**		
ST6GAL1 (T227P)		CYB561 (A242T)	SPPL2C (S542L)	PARP9 (C696G)		
TTN (N22495K)		***DICER1 (P1377H)***	SQSTM1 (A83T)	PI4KB (S254*)		
VWF (Q2064K)		DYNC2H1 (R82G)	TAF4B (G10V)	PKHD1L1 (E967*)		
		EPPK1 (G1575A)	TBC1D22B (I482T)	PM20D1 (K344*)		
		ERN1 (D145Y)	TLR10 (G282V)	PPM1B (Y205*)		
		FOXK2 (S30A)	TSPEAR (D117G)	PRSS56 (S64*)		
		IARS2 (N768D)	UBR4 (P2206S)	PTPRN (P195S)		
		IFFO1 (M561R)	UNC45B (E11D)	S100A11 (R62H)		
		ISM1 (L168F)	WNT8B (Q71K)	SH3GL3 (R294M)		
		KIF6 (Q677K)	XPO6 (R699*)	SS18L1 ( S107F)		
		MAST4 (M1431I)	YDJC (R220H)	ST5 (L146F)		
		MS4A14 (L139F)	ZNF646 (R677W)	STXBP6 (L126F)

The number of somatic mutations at time of diagnosis was correlated with patient age at time of sampling, at time of both diagnosis and relapse/progression. Pearson correlations were calculated after removing case NB67R9, which appears to have had a markedly higher mutation rate than the other samples (Fig. 1C). For the remaining tumor specimens, Pearson correlation coefficients indicate statistically significant correlation between the total number of non-synonymous SNVs and patient age at time of diagnosis or relapse occurrence (*R* = 0.915, *p* = 3.0 × 10^–5^) (Fig. 1C).

### Copy number alterations and structural variants

**Figure 2:**
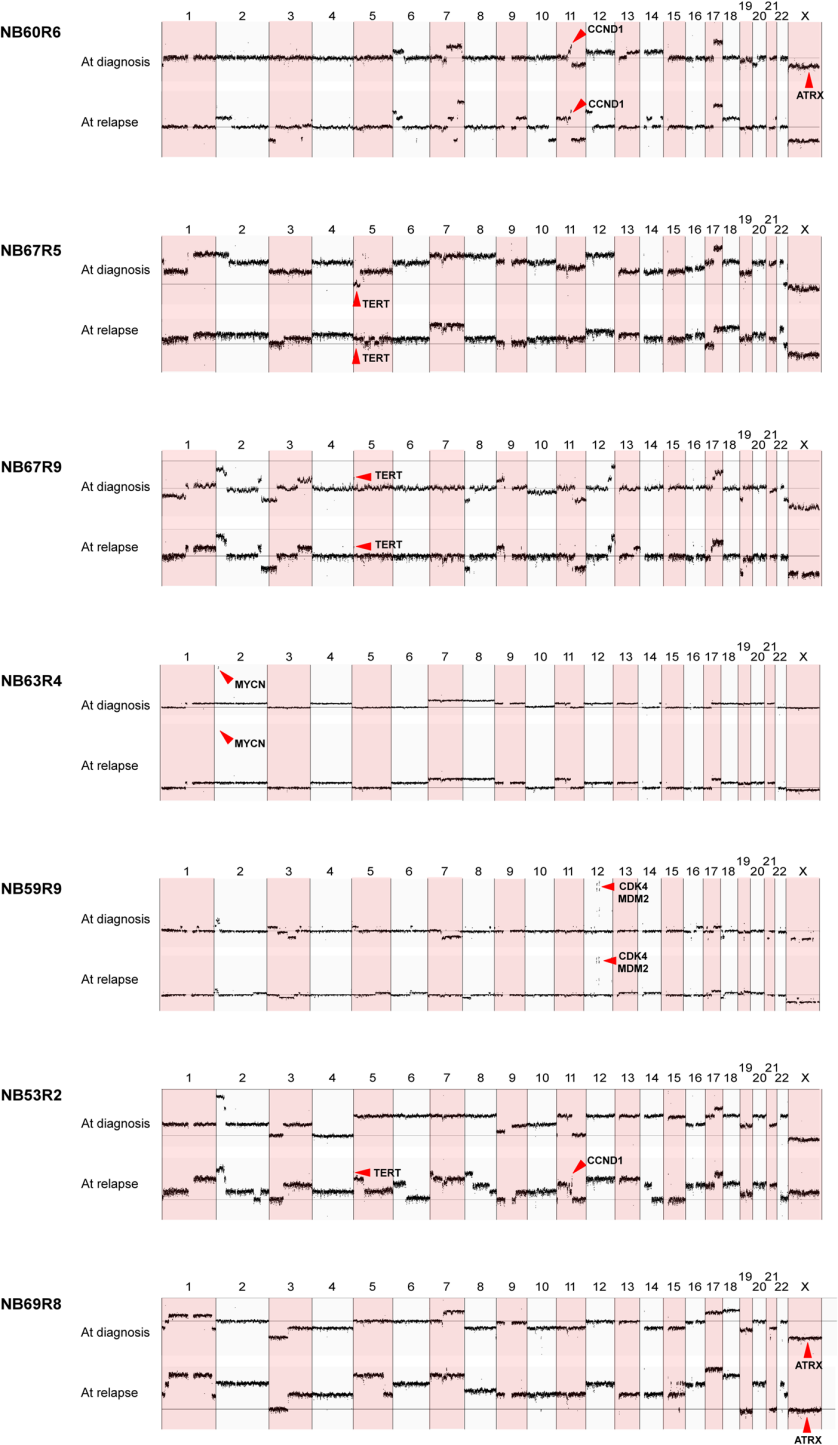
Copy number aberrations. Coverage-based plots displaying segmental alterations in primary and relapsed tumors.

Although some persistent copy number alterations (CNAs) were detected in matched primary and relapsed tumors, the relapsed tumors had increased numbers of structural variants (SVs) at time of relapse (Fig. 1B, Supplemental Fig. 1, and Supplemental Table 4). At time of diagnosis, a median of 38 somatic SVs (range 5–122) were detected, the number later increasing to a median of 49 SVs (range 14–89) at time of relapse when excluding the excessive number of SVs within highly amplified regions (i.e., the *MYCN* locus in NB63R4 and the 12q-amplicons in NB59R9). Persistent CNAs present in both diagnostic and relapsed/refractory tumors include: 17q-gain, detected in all tumors except one that instead displayed gain of whole chromosome 17; 11q-deletion, detected in four of six patients; *MYCN* amplification, detected in one patient; and *CDK4/MDM2/FRS2* amplification, detected in one patient (Fig. 2 and Supplemental Fig. 1). Loss of chromosome 1p, also associated with poor prognosis in NB^21^, was seen in six patients: three cases (i.e., NB67R9, NB63R4, and NB69R8) in which 1p-deletion was detected in both primary and relapse tumors, one case (i.e., NB60R6) in which 1p-deletion was detected only in the diagnostic specimen, one case (i.e., NB53R2) in which 1p-deletion was detected only in the relapsed specimen, and one case (NB67R5) in which 1p-deletion was detected in both primary and relapsed tumors, albeit with different breakpoints (Fig. 2 and Supplemental Table 4). On the genomic level, additional recurrent CNAs and structural variants were seen in association with *TERT* (in three patients, one relapse specific), *CCND1* (in two patients, one relapse specific), and *LSAMP* (in two patients, both relapse specific) (Fig. 1D,E). Interestingly, both *TERT* SVs in patient NB67R5 and the *CCND1*-gains in patient NB60R6 appear to have emerged on separate occasions as parallel events, given the difference in translocation partners and breakpoints relating to these genomic alterations (Supplemental Table 4). In patient NB60R6 we also detected CNAs in both primary and relapse tumors, resulting in gain of chromosomal regions 7q and 6p; however, this also emerged through parallel events, as the two tumors have different breakpoints for these CNAs and also gains of different parental chromosomes as judged by the allele fraction (Figs. 1D and 2). The paired primary and relapse tumors of patient NB60R6 shared only one common SV, a translocation between chromosomes 11 and 17 that ultimately resulted in 11q-deletion and 17q-gain. However, as the position of the translocation was identical in both samples, this indicates that both analyzed specimens share a common descent. Patient NB59R9 had the highest number of SVs due to high genomic instability, with a cataclysmic event resulting in extensive focal genomic shattering and rejoining. This affected regions in chromosome 5 and amplified regions in chromosome 12, connected to *MDM2* and *CDK4*, were present in both samples for this patient, while similar focal genomic shattering was seen in chromosomes 7, 18, and 21 in primary tumors and in chromosome 4 in relapse tumors (Supplemental Table 4).

### Recurrent alterations and mutations in cancer-associated pathways

Telomere maintenance is crucial for sustained proliferative capacity and is associated with poor prognosis in NB. In our cohort, tumors from three patients had *TERT*-associated SVs and tumors from two patients had tandem duplications causing disruption of *ATRX* (Fig. 1D,E). All three patients with *TERT* alterations had genomic profiles that included 11q deletions, though in two patients we did not detect any alterations in genes directly connected to telomere maintenance. Both these patients displayed high levels of genomic amplification: patient NB59R9 for several 12q loci, including *CKD4*, *MDM2*, and *FRS2*, and patient NB63R4 for the *MYCN* locus. Interestingly, patient NB67R5, with two different *TERT*-associated structural variants in primary and relapsed tumors, also had two mutations in the chromatin-associated genes *ARID1A* and *ARID1B*. The primary tumor of NB67R5 had a structural variant causing disruption of *ARID1B*, a variant that was lost in the relapse sample, which instead carried a missense mutation in *ARID1A* (Fig. 1D, and Supplemental Tables 2 and 3).

Previous studies by us and others have indicated that mutations in *ALK* and other genes of the RAS-MAPK pathway are connected to relapsed NB^18,19^. In our cohort, an activating somatic *ALK* mutation was seen in the tumors from one patient (i.e., NB69R8). This is an ALK Q1275R alteration in which the tumor displayed increased variant allele frequency from time of diagnosis to time of relapse. This increase was likely due to a new copy-neutral loss of heterozygosity (CN-LOH) of chromosome 2 that preserves the mutated allele, as judged by B-allele frequency. In addition to *ALK*, somatic variants were also detected in other genes associated with RAS-MAPK signaling. This includes mutations in *KRAS*, *FGFR1*, and *MAP3K14* (NIK), the first two genes being detected only in the relapsed samples of two different patients, while the last was shared by the primary and relapse tumors in one patient (Fig. 1D). We also detected a somatic frameshift mutation in *PHOX2B*, a gene important for proper neural crest development and present in both the primary and recurrent tumors of patient NB67R5. Somatic alterations were seen in several genes connected to cell cycle progression and/or DNA repair, such as *CCND1*, *CDK4*, *MDM2, BRCA1*, *BRINP1*, and *WRN* (Fig. 1D).

Besides *CCND1* and *TERT*, 30 additional genes were altered in at least two tumor samples in our cohort, either through SNV or SV. Recurrent mutated genes present only in tumors at time of diagnosis were limited to *ABCA5*, *CNTNAP2*, *CTNND2*, and *PTPRM*, while we detected recurrent mutations in relapsed samples of 16 genes either already present in primary tumors or acquired in relapse. Of these 16 genes, eight were found to be relapse specific (i.e., *CDK5RAP1*, *CFAP44*, *LSAMP*, *MUC16*, *NUBPL*, *POLR2A*, *PRR12*, and *SLC6A18*) (Fig. 1D and Table 3). Two tumors carried missense mutations predicted to be damaging by SIFT and PolyPhen2 in the tumor-suppressor gene *FAT1* (Fig. 1D). The *FAT1* mutation in NB63R4 was acquired during relapse, while the *FAT1* mutation in NB67R5 was present at the subclonal level with a 2% variant allele fraction in the primary tumor but increasing in relapse (i.e., a 15% variant allele fraction), indicating clonal expansion (Supplemental Table 3 and Supplemental Fig. 2).

### Pathway-centered gene set enrichment of relapsed and primary neuroblastoma

Pathway analyses were performed on somatically mutated genes detected among all relapsed and primary tumors, respectively, to detect differences in pathway signature. Gene set analyses in KEGG for all mutations present in relapse tumors identified pathways connected to the glutamatergic synapse, PI3K-Akt signaling, longevity regulation, and Rap1 signaling among the highest ranked enrichment although not at a statistically significant level after correction for multiple testing (Supplemental Fig. 3 and Supplemental Table 5). Gene set analyses also indicated the prior enrichment of genes involved in PI3K-Akt signaling and in cancer pathways in the diagnostic samples (Supplemental Fig. 3 and Supplemental Table 5).

**Figure 3:**
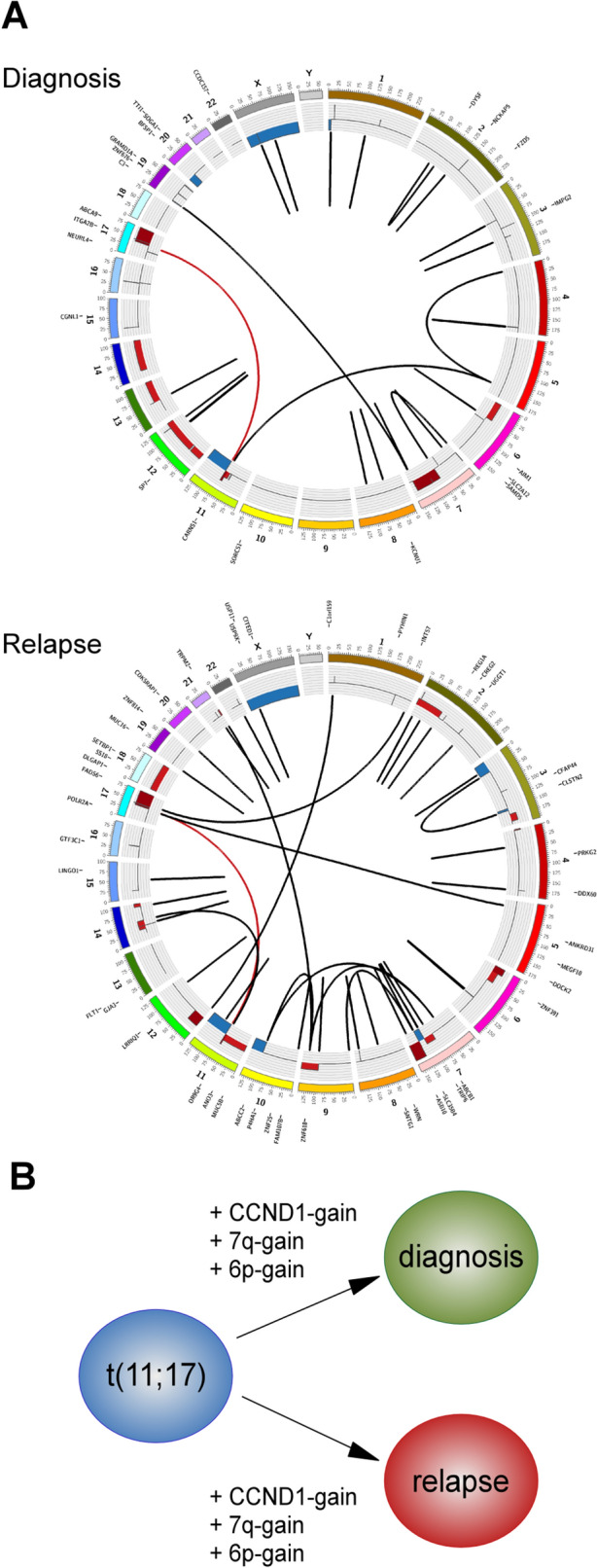
Broad patient-specific tumor heterogeneity with converging genomic alterations. (**A**) Striking tumor heterogeneity is seen between tumor material from time of diagnosis and time of relapse in patient NB60R6. Only one SV, an unbalanced translocation between chromosomes 11 and 17, is shared by the two samples. (**B**) The two samples display converging genomic events, such as gain of *CCND1*, 7q, and 6p, albeit with different breakpoints and different parental alleles.

## Discussion

In attempting to identify molecular features related to disease progression in NB, we have characterized the genomes of seven relapsed/progressive NB tumors and corresponding tumor specimens from the time of diagnosis. In this study, we show that relapsed tumors generally have a higher mutational load and have acquired additional segmental variants (Fig. 1A,B, and Supplemental Fig. 1), in line with previous studies of relapsed NB^19,20^. We also show that the number of mutations present in primary tumors and the number of acquired mutations in relapsed tumors correlates well with patient age at diagnosis and recurrence, respectively (Fig. 1C). The coverage depth used for WGS in this study limits the ability to analyze whether relapse-specific mutations were acquired de novo or emerged from subclonal events in the primary tumor. However, we do detect examples of the clonal expansion of specific mutations, for example, as seen for *FAT1* (Supplemental Fig. 2), as well as the presence of clonal eradication, as only a mean of 36% of all mutations detected in primary tumors are also present in relapse tumors. For some samples, such as NB60R6 and NB67R9, there is striking in-patient heterogeneity, with very few somatic SNVs shared by different tumor samples (Fig. 1E). This indicates that the longitudinal monitoring of disease burden and treatment response using specific genetic alterations could be challenging for a subgroup of NB patients.

Recurrent alterations were seen in 32 genes in tumors from at least two patients; eight of these genes were relapse specific and include *MUC16* and *LSAMP* (Fig. 1D). Tumors from three patients also display alterations connected to cell cycle genes. These include tumors from two patients with *CCND1* gain and tumors from one patient with *CDK4/MDM2* amplification (Fig. 2).

Mutations in *ALK* have previously been shown to be enriched in relapsed NB^18^, as have other mutations that activate RAS-MAPK signaling, such as *RAS*, *NF1*, *BRAF*, and *FGFR1*^19^. Tumors from four of our seven patients carry somatic ALK-MAPK-RAS signaling pathway alterations. These include somatic mutations in *ALK* and *MAP3K14* that were already present at time of diagnosis and mutations in *KRAS* and *FGFR1* that are relapse specific (Fig. 1D). The *KRAS* K117N alteration, detected in patient NB67R5, has been shown to decrease GTPase activity^22,23^ although studies from colorectal cancer indicate this alteration to be less potent than mutations in codons 12 and 13^22^. The tumor with KRAS K117N also carried a *FAT1* mutation present at the subclonal level in the primary tumor and later found to be enriched at time of relapse. *FAT1* is a tumor-suppressor gene that restrains the oncogenic activity of the Yes-associated protein (YAP)^24^, and studies of other malignancies show that YAP is a critical effector of oncogenic RAS^25,26^. Therefore, it is possible that the malignant capacity of KRAS K117N is further potentiated through increased YAP signaling in relapsed tumors.

Both *PHOX2B* and *ALK* have previously displayed recurrent mutations in both familial and sporadic NB^4,5,27^, as they are crucial for proper development of the sympathetic nervous system. In our cohort, the tumors from one patient (i.e., NB67R5) shared a somatic frameshift mutation of *PHOX2B* (p.G230fs). Truncating *PHOX2B* mutations have been shown to possess a dominant-negative effect resulting in halted sympathetic nervous system differentiation and increased proliferation, making cells susceptible to additional transforming events^28^. Regarding *PHOX2B* and *ALK* alterations in constitutional DNA, we detected a novel *ALK* frameshift variant already present in the germline (p.V1471fs*6) in one investigated patient. Although *ALK* is of great interest in NB etiology, it is difficult to envision the role of this particular variant in the oncogenic process, as it is expected to lack phosphorylation sites essential for ALK activation.

However, it has been shown that children and adolescents with cancer have a higher incidence of pathogenic or likely pathogenic germline mutations than do healthy controls^29^. When expanding our investigation of other cancer predisposition genes besides *ALK* and *PHOX2B*, we detect rare variants such as a nonsense mutation in *BARD1* as well as missense variants in *CHEK2*, *BRCA2*, and *WRN* (Table 2). Germline variants in *BARD1* and *CHEK2* have indeed been shown to be enriched in NB patients^16,30^, while *BRCA2* has been shown to be enriched in pediatric cancer patients^29^. The gene *WRN* encodes a RecQ helicase important for maintaining genome stability through resolving stalled replication forks in association with double-strand breaks. The missense variant detected in *WRN* affects an ATM phosphorylation site required for correctly handling stalled forks^31^. The tumors of patients who are heterozygous for the *WRN* variant display high genomic instability, with extensive focal genomic shattering and rejoining that might be attributed to deficient double-strand break repair.

For one patient (i.e., NB60R6), we analyzed a metastasis at time of diagnosis and a metastasis at time of relapse that provided indications of branched evolution. These two tumor specimens displayed no common nonsynonymous SNVs and only one shared structural variation, a translocation between chromosomes 11 and 17 resulting in 11q-deletion and 17q-gain (Fig. 3A). That the fusion point was exactly the same in both samples indicates that they emerged from a common ancestral tumor cell progenitor and not through multifocal events. However, both lesions acquired 6p-gain, 7q-gain, and *CCND1*-gain, albeit with different breakpoints and gains of different parental alleles (Figs. 2 and 3B). Additional parallel events between primary and relapse tumors were seen in case NB67R5, which displayed two different SVs close to the *TERT* locus, and in case NB67R9, which displayed two different mutations in *ARID1B* and *ARID1A*, two members of the SWI/SNF chromatin-remodeling complex, in tumors at time of diagnosis and time of relapse, respectively (Fig. 1D). The primary and relapsed tumors from patient NB67R9 also had a common *TERT* rearrangement that might be further potentiated through the *ARID1B* and *ARID1A* mutations, as loss of *ARID1A* has been shown to enhance *TERT* transcription^32^.

The findings of alterations of *TERT*, *ARID1A*, *and ARID1B* are related to telomere maintenance, which is a strong predictor of poor outcome in NB^13,33^. Besides the *MYCN*-amplified NB case, in which sustained telomere length might be achieved through elevated *TERT* expression driven by *MYCN*, all six non-*MYCN*-amplified cases in our cohort displayed genomic aberrations associated with telomere maintenance or chromatin remodeling; three of these cases had *TERT* rearrangements, two had *ATRX* alterations, and one had a mutation in *ARID2*, which is also part of the SWI/SNF chromatin-remodeling complex. This further supports the finding that telomere maintenance is associated with an unfavorable clinical course in NB^13,33^, as most tumors in this study were characterized by different means of sustaining telomeres.

In this study we have identified a diverse set of gene mutations in tumors from time of diagnosis and time of recurrence in this challenging patient group. This set includes mutations in genes promoting cell cycle progression, activating ALK-RAS-MAPK signaling, or important for telomere maintenance and immortalization. Our findings in combination with previous studies of relapsed NB provide a rationale for the comprehensive genomic characterization of recurrent NB in order to optimize treatment and aim for prolonged survival. This information could be used to spur novel combination therapies using compounds abrogating MAPK signaling, cell cycle progression, and telomere activity.

## Material and methods

### Patient material and ethics

NB tumors were collected from Swedish patients after either written or verbal informed consent was obtained from parents/guardians according to ethical permits approved by the local ethics committee (Karolinska Institutet and Karolinska University Hospital, registration numbers 03-736 and 2009/1369-31/1) in accordance with the Declaration of Helsinki. Clinical data on all patients were obtained from hospital records and/or the Swedish Childhood Cancer Registry.

### Whole-genome sequencing

DNA was extracted from frozen tumors or blood using the DNeasy blood and tissue kit (Qiagen, Hilden, Germany) according to the manufacturer’s protocol and evaluated using absorbance measurements, fluorometric quantitation, and DNA integrity assessment on an Agilent TapeStation (Agilent, Santa Clara, CA) before sequencing. WGS was performed on triplicate samples of DNA from tumor material at time of diagnosis, tumor material at time of relapse/refractory disease, and corresponding constitutional DNA extracted from the blood of seven NB patients. For TruSeq DNA PCR-Free library preparation, 1 µg of DNA was used for all samples except for the primary tumor from NB59R9, for which a modified low-input protocol using 100 ng of DNA was required due to limited material. WGS was performed aiming for an average coverage of at least 60X for tumor libraries prepared according to the PCR-free library protocol and 30X for constitutional DNA and tumor samples prepared according to the low-input protocol using Illumina xTen instrumentation (Illumina, San Diego, CA, USA) located at Clinical Genomics, SciLife Laboratories, Stockholm, Sweden. Details of sequencing coverage are given in Supplemental Table 1. Mapping to the human reference genome hg19, removal of read duplicates, realignment around InDels, and base-score recalibration and variant calling were carried out using the Sentieon suite of bioinformatics tools (Sentieon Inc., Mountain View, CA, USA). This included the variant calling of germline single nucleotide variants (SNVs) and small InDels (up to about 80 bp in size) using Sentieon DNAscope software, while Sentieon TNscope was used for the calling of corresponding somatic variants, supplying the normal sample to filter out germline variants. In both DNAscope and TNscope variant calling, the corresponding machine learning models developed by Sentieon were used to filter out likely artifacts (Sentieon version v201808.03). Only high-quality called variants with a minimum 10% variant allele frequency and a total read coverage of ten were considered for further analysis. All synonymous variants or variants in non-coding regions were excluded, except those affecting canonical splice sites and remaining variants were assessed manually using the Integrative Genomics Viewer (IGV)^34^ to remove calls due to mapping artifacts and paralogs. The germline variants were analyzed using a more stringent filtering approach excluding all variants with a population allele frequency above 1.0%, in either gnomAD v2.1.1 (https://gnomad.broadinstitute.org/), 1000 genomes, the Exome Aggregation Consortium (ExAC) (Cambridge, MA, USA, http://exac.broadinstitute.org), or the NHLBI Exome Sequencing Project (http://evs.gs.washington.edu/EVS/), unless being an already established pathogenic variant, and keeping only nonsynonymous variants of genes selected based on established connections to hereditary and/or sporadic cancer genes, as listed in Supplemental Table 2. The filtering of somatic and germline variants was done using the ingenuity variant analysis software v5.6 (Qiagen, Hilden, Germany).

The Canvas tool (version 1.38.0.1554)^35^ was used to call copy number alterations (CNAs), with gains and losses of genomic regions as well as LOH regions being identified using read depth coverage in combination with SNV b-allele frequencies. For the calling of germline and somatic CNVs, the SmallPedigree-WGS and Somatic-WGS algorithms of Canvas were used, respectively. Somatic structural variants (SVs) were called using the Manta tool (version 1.1.1)^36^, which applies information about soft-clipped read ends, disjointed read pairs, and read-pair orientation to identify larger structural variations (e.g., deletions, duplications, inversions, and translocations). Calls from the constitutional DNA were used to filter out germline variation and artifacts caused by problematic regions. In addition, further filtering and removal were done based on presence in the SweGen Variant Frequency dataset (https://swefreq.nbis.se/) or in our in-house set of normal controls. Genes affected either directly by SVs or through smaller focal gain/loss were also noted for further genomic analysis.

To verify same individual origin for matched tumor-normal pair, we ran a Python script developed in-house (available on request) that calculated the fraction of shared single nucleotide polymorphisms (SNPs) relative the total number of SNPs using 400,000 SNPs (all present in dbSNP; version 138) from chromosomes 1–20. Using this method, the fraction reliably ends up in the 0.97–1 range for verified tumor-normal matched pairs, whereas unrelated randomly selected samples have a fraction between 0.6 and 0.7, while a parent and child-level of relatedness ends up close to 0.8. All paired samples in this study showed a tumor-normal matched fraction above 0.97 and thus, authenticated sample identity.

### Statistics

IBM SPSS software was used to calculate Pearson correlations between mutational loads at diagnosis and relapse, respectively, relative to age at time of sampling and to perform independent *t*-testing for differences in the number of somatic SNVs between primary and relapsed tumors. Gene set analysis for the enrichment of somatically mutated genes was conducted using the curated Kyoto Encyclopedia of Genes and Genomes (KEGG) cell signaling pathway database^37^ (www.kegg.jp/kegg/kegg1.html) through the Enrichr web server^38^.

## Supplementary information

Supplementary Figures.

Supplementary Tables.
