# Supplementary material for: Whole-genome sequencing of recurrent neuroblastoma reveals somatic mutations that affect key players in cancer progression and telomere maintenance
Source: Sci Rep. 2020 Dec 31;10:22432. doi: 10.1038/s41598-020-78370-7 (PMC7775426; doi:10.1038/s41598-020-78370-7)
Supplement: Supplementary file 1 — Supplementary Figures. [file 41598_2020_78370_MOESM1_ESM.pdf]

Supplemental figures

Whole-genome sequencing of recurrent neuroblastoma reveals somatic mutations that affect key players in cancer progression and telomere maintenance

Susanne Fransson[1*], Angela Martinez-Monleon[1], Mathias Johansson[2], Rose-Marie Sjöberg[1], Caroline Björklund[3], Gustaf Ljungman[4], Torben Ek[5], Per Kogner[6§] and Tommy Martinsson[1§]

[1]Department of Laboratory Medicine, Institute of Biomedicine, University of Gothenburg, Gothenburg, Sweden.
[2]Science for Life Laboratory, Clinical Genomics, Gothenburg, Sweden.
[3]Department of Pediatrics, Umeå University Hospital, Umeå, Sweden.
[4]Department of Women's and Children's Health, Children's University Hospital, University of Uppsala, Uppsala, Sweden.
[5]Children's Cancer Center, Queen Silvia Children's Hospital, Sahlgrenska University Hospital, Gothenburg, Sweden.
[6]Department of Women's and Children's Health, Karolinska Institutet, Stockholm, Sweden.

Supplemental Figure 1

At diagnosis	At recurrence

NB60R6

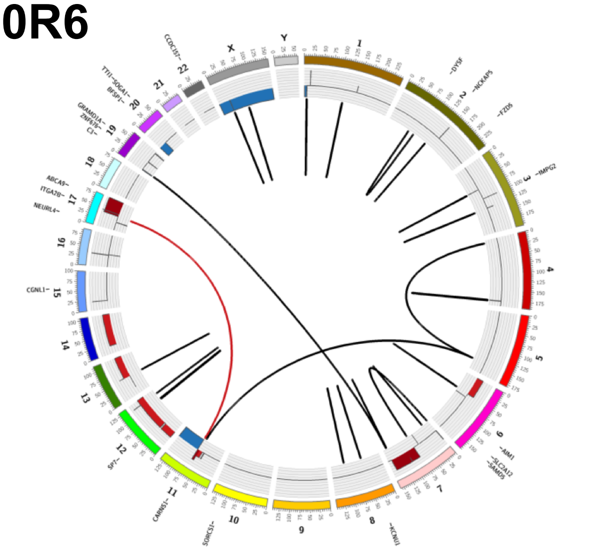

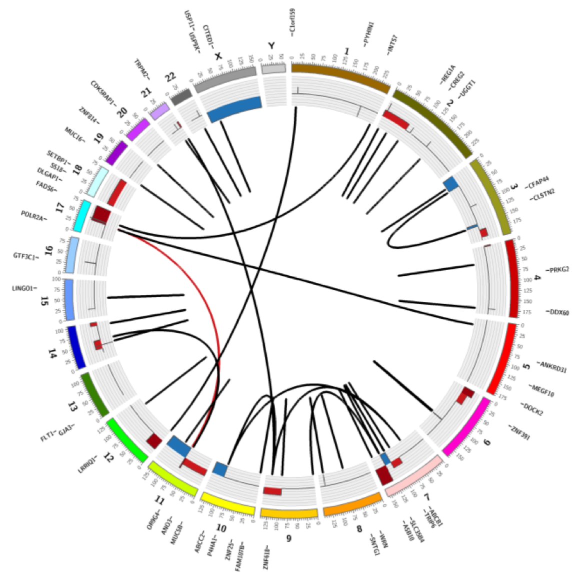

NB67R5

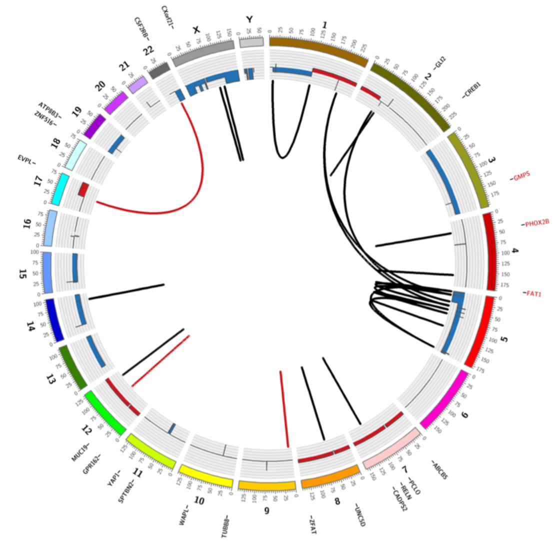

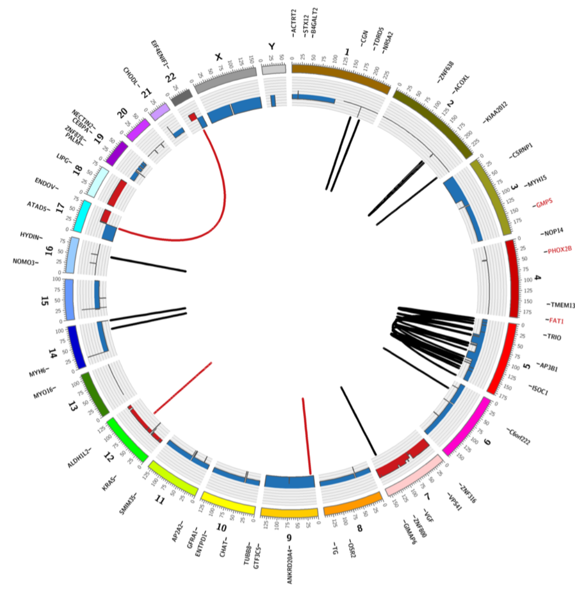

NB67R9

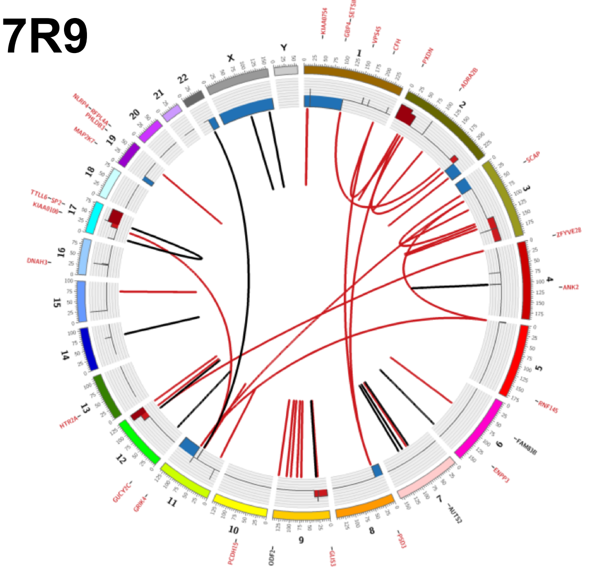

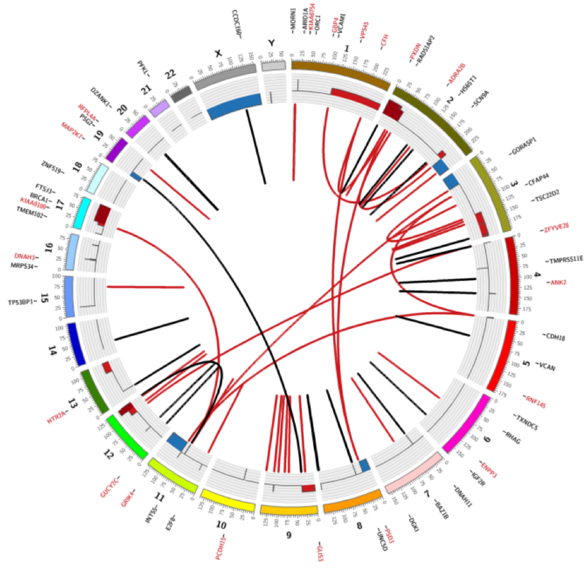

Supplemental Figure 1 - continued

	At diagnosis	At recurrence
**NB63R4**	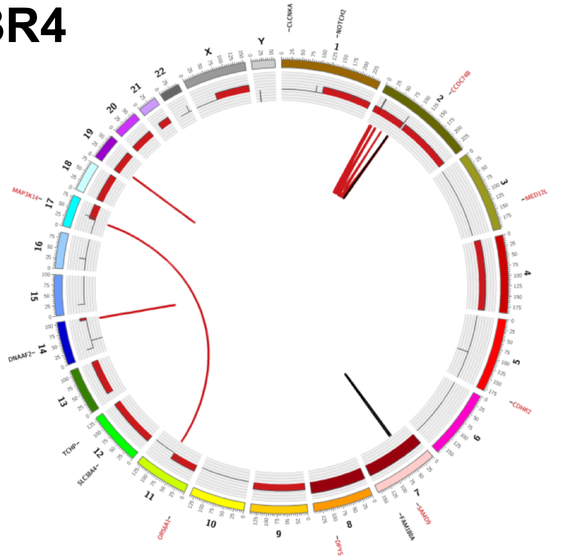	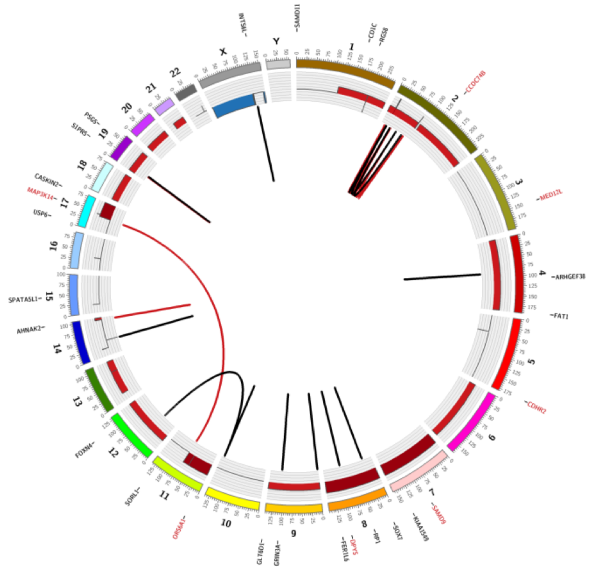
**NB59R9**	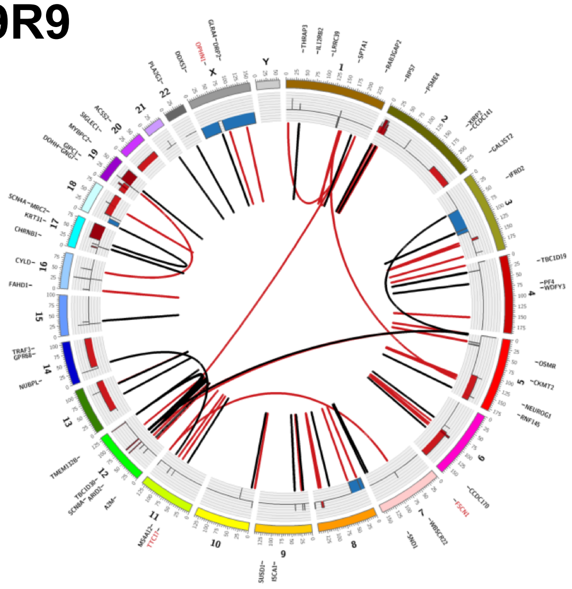	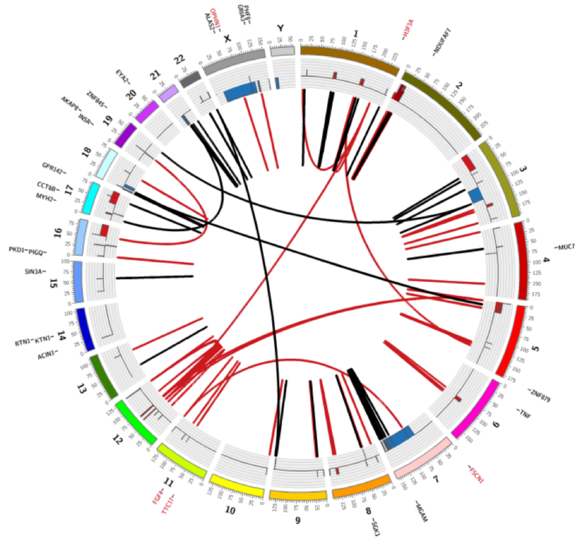
**NB53R2**	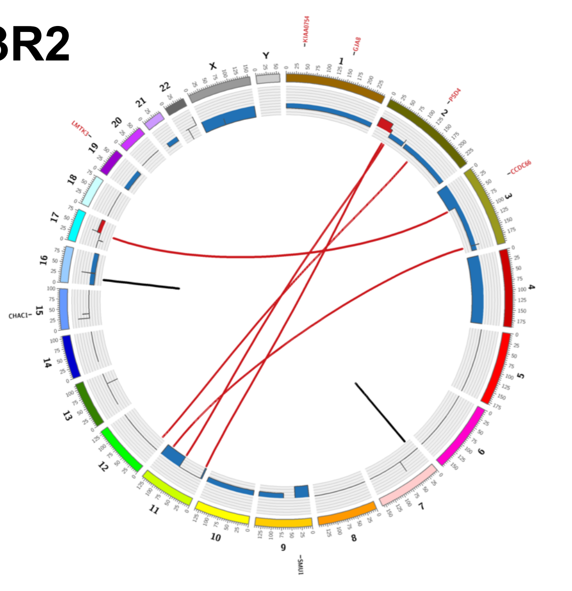	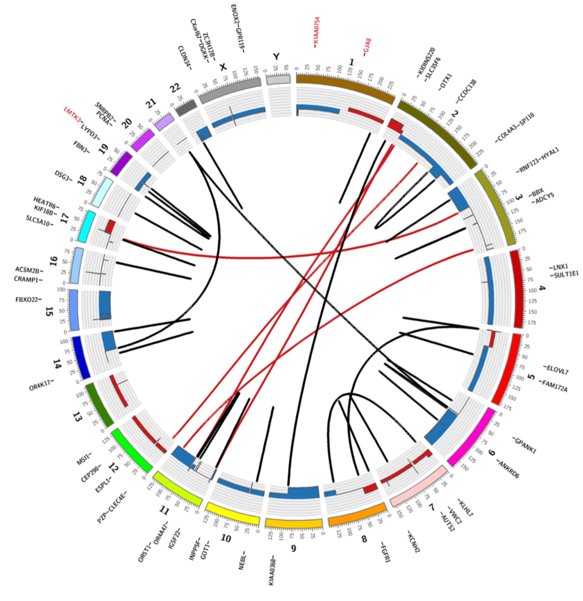

Supplemental Figure 1 - continued

At diagnosis   At recurrence

NB69R8

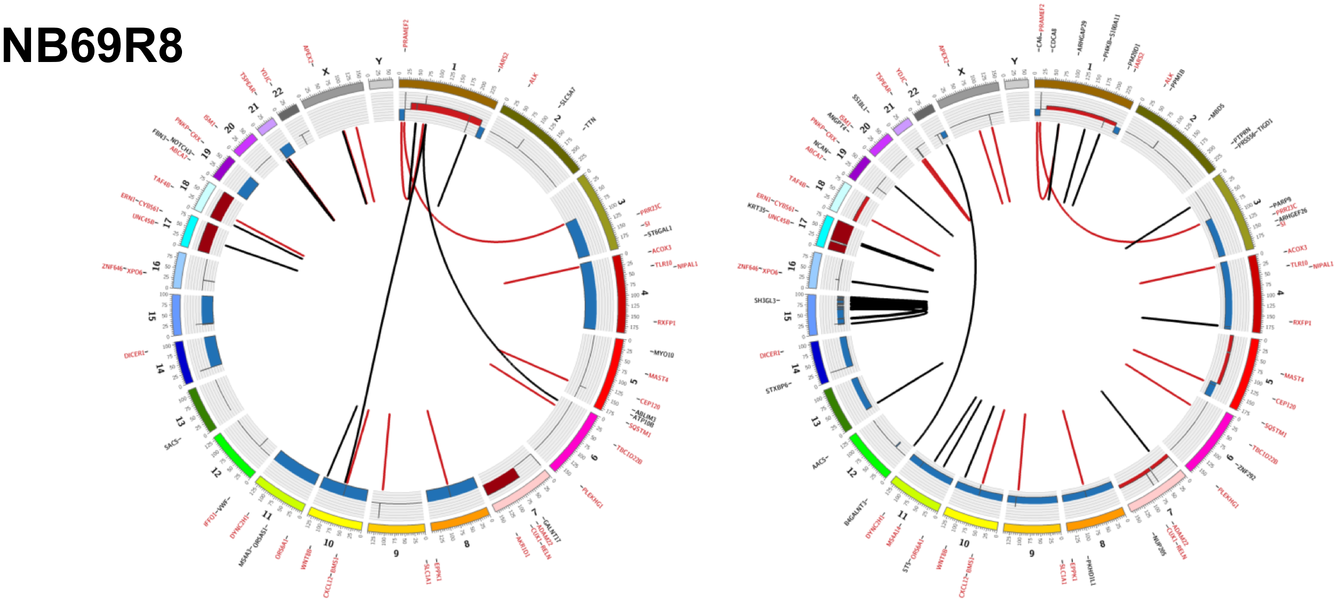

Supplemental Figure 1. Mutational spectra of diagnostic and relapsed NB tumors. Circos plots showing structural variants, copy number alterations, and somatic SNVs. Copy number plots calculated based on the coverage ratio between tumor and corresponding normal tissue are shown on the inner circle, with gain of genomic material indicated in red and loss of genomic material indicated in blue. The lines within the inner circle indicate structural variants within and between chromosomes, while genes affected by somatic SNVs are shown outside the outer circle. Aberrations are colored according their presence: black aberrations are unique to the respective samples, while red aberrations are shared by tumor material at time of diagnosis and at time of relapse.

Supplemental Figure 2

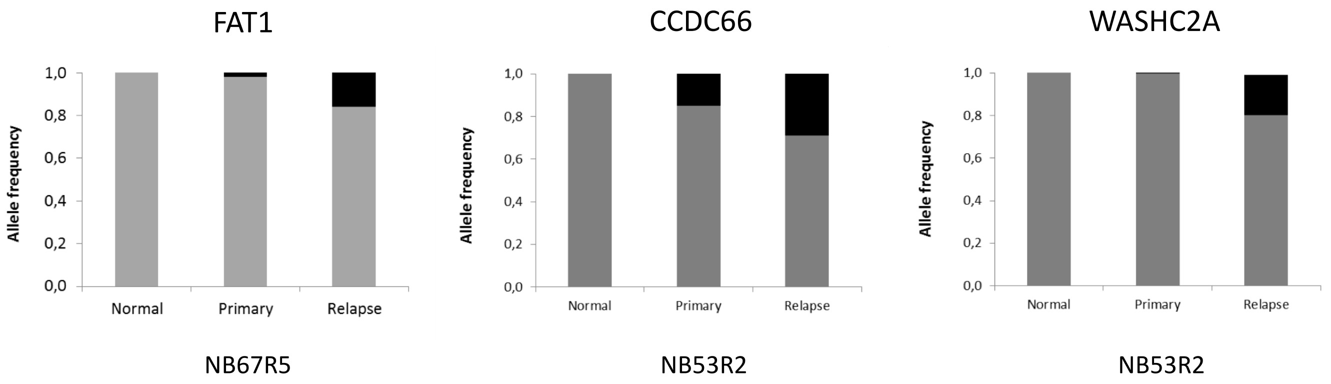

Supplemental Figure 2. Allele frequency increase shifts in relapse tumors. A shift from low allelic frequency in primary tumors to significant enrichment in corresponding relapse tumors is seen in *FAT1*, *CCDC66*, and *WASH2A*.

Supplemental Figure 3

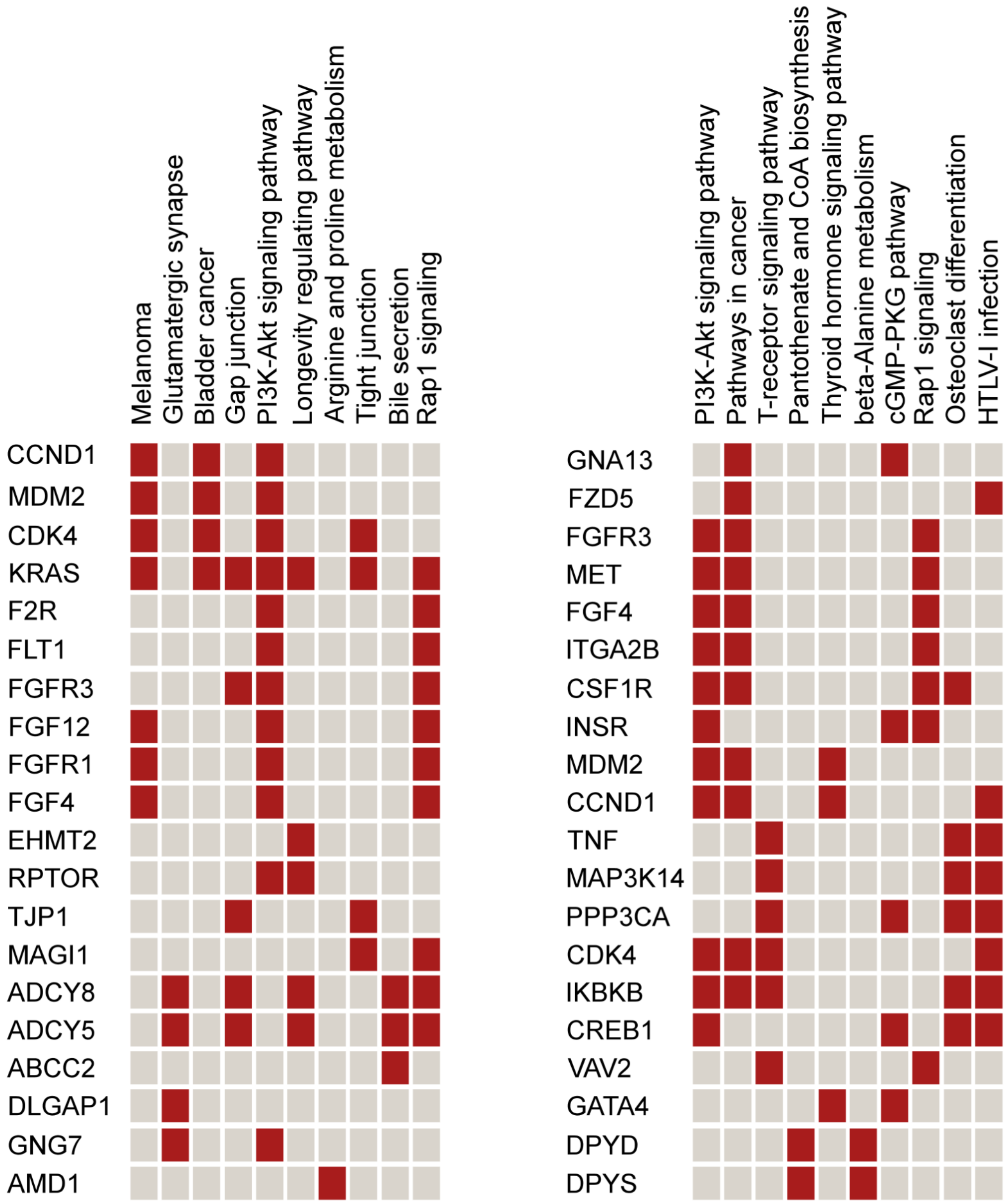

Supplemental Figure 3. Mutational pathway enrichment analysis. Gene set enrichment analysis in curated KEGG pathways of somatically altered genes in all relapsed samples (left panel) and all diagnostic samples (right panel).
